# Role of DUSP1/MKP1 in tumorigenesis, tumor progression and therapy

**DOI:** 10.1002/cam4.772

**Published:** 2016-05-26

**Authors:** Jiliang Shen, Yaping Zhang, Hong Yu, Bo Shen, Yuelong Liang, Renan Jin, Xiaolong Liu, Liang Shi, Xiujun Cai

**Affiliations:** ^1^ Department of General SurgerySir Run‐Run Shaw HospitalZhejiang University, School of MedicineHangzhou310016China; ^2^ Department of AnesthesiologySir Run‐Run Shaw HospitalZhejiang University, School of MedicineHangzhou310016China

**Keywords:** Carcinogenesis, DUSP1/MKP1, JNK, tumor therapy

## Abstract

Dual‐specificity phosphatase‐1 (DUSP1/MKP1), as a member of the threonine‐tyrosine dual‐specificity phosphatase family, was first found in cultured murine cells. The molecular mechanisms of DUSP1‐mediated extracellular signal‐regulated protein kinases (ERKs) dephosphorylation have been subsequently identified by studies using gene knockout mice and gene silencing technology. As a protein phosphatase, DUSP1 also downregulates p38 MAPKs and JNKs signaling through directly dephosphorylating threonine and tyrosine. It has been detected that DUSP1 is involved in various functions, including proliferation, differentiation, and apoptosis in normal cells. In various human cancers, abnormal expression of DUSP1 was observed which was associated with prognosis of tumor patients. Further studies have revealed its role in tumorigenesis and tumor progression. Besides, DUSP1 has been found to play a role in tumor chemotherapy, immunotherapy, and biotherapy. In this review, we will focus on the function and mechanism of DUSP1 in tumor cells and tumor treatment.

## Introduction

Dual‐specificity phosphatase‐1 (DUSP1, also called MKP‐1, ERP, CL100, HVH1, PTPN10, and 3CH134) was initially identified in cultured murine cells 1, 2. It has two domains consisting of an amino terminal noncatalytic domain and a C‐terminal catalytic domain (Fig. S1), which contains the ATPase active site consensus sequence 3, 4. DUSP1 is one member of dual‐specificity phosphatases which are recognized as key players for inactivating different mitogen‐activated protein kinase (MAPK) isoforms 5, 6, 7. The members of MAPK family, including extracellular signal‐regulated protein kinases (ERKs), JNKs, and p38 MAPK, are reported to play important roles in cell proliferation and apoptosis. In general, the ERK1/2 cascade appears to mediate signals promoting cell proliferation, differentiation, or survival, whereas, the JNK and p38 MAPK cascades appear to be involved in the cell responses to stresses. DUSP1 obtains the ability to inactivate ERK, JNK, and p38 in vivo by dephosphorylation process 8, 9, 10, 11. As the archetypal member of its family, DUSP1 has been exhaustively studied. DUSP1 plays a role in cell proliferation, differentiation and transformation, stress responses, inflammation, cycle arrest, and apoptosis mainly by regulation of MAPK signaling 12, 13, 14. The level of DUSP1 was also investigated in human tumors. These initial studies revealed a higher DUSP1 expression in a range of human epithelial tumors including prostate, colon, and bladder, however, the expression of DUSP1 in tumors decreased progressively with a higher histological grade 15, 16, 17.Some studies also suggested that DUSP1 was involved in tumor therapy efficiency 18, 19, 20, 21, 22. The function and mechanism of DUSP1 in tumors may be varied and complex. Therefore, in this review, we will focus on the function and mechanism of DUSP1 in tumorigenesis, tumor progression, and antitumor therapy.

## Role of DUSP1 in Carcinogenesis

Imbalance between cellular proliferation and death induced by apoptosis, with decrease in some proapoptotic signals and upregulation of some antiapoptotic signals, is the characteristic of carcinogenesis 23. The role of DUSP1 in carcinogenesis could be controversial in different tumors.

### DUSP1 promotes carcinogenesis

DUSP1 expression was found to be upregulated in prostatic cancer compared with normal tissues. Further study suggested that DUSP1 may promote prostatic carcinogenesis via inhibiting Fas/FasL‐induced cell apoptosis 24, 25. Parallel with the prostate cancer, a similar function of DUSP1 was found in pancreatic cancer, as immunohistochemistry revealed an 8.1‐fold increase in mean DUSP1 mRNA level in pancreatic cancer tissues compared with normal pancreatic tissues. Knocking down DUSP1 in pancreatic cancer cells attenuated the tumorigenicity in a nude mouse model 26. Increased expression of DUSP1 was also observed in other tumors, including colon, bladder, gastric, breast, and lung cancer 15, 16, 17, 27, 28. As a result of DUSP1 increase, JNK activation would be inhibited which consequently protect tumor cells from JNK‐induced apoptosis (Table.1).

**Table 1:** Different roles of DUSP1 in different kinds of tumors

Cancer	Tumor/Normal expression	Carcinogenesis	Main pathway/genein carcinogenesis	DUSP1 in tumor withhigher histological grade
Prostate, pancreas, colon, ovary, bladder, gastric [15–17,24,27,28]	Increased	Promote	JNK	Decreased
Lung [28,40]	Increased	Promote	JNK	Decreased, its role in tumor progression is inconclusive
Hepatocellular carcinoma [29–33]	Decreased	Inhibit	Hcr	Decreased
Head and neck squamouscell carcinoma [34,35]	Decreased	Inhibit	IL1 beta	Unknown

### DUSP1 inhibits carcinogenesis

The expression of DUSP1 in hepatocellular carcinoma (HCC) decreased slightly compared with normal liver tissues. Tsujita E 29 reported that 11/77 patients were detected lower levels of DUSP1 in tumor tissues in comparison with the levels in normal tissues, but no big difference in another 66 patients. Nevertheless, little change of DUSP1 expression was found between HCC tissues and normal liver tissues. DUSP1 was testified to play a role in inhibiting hepatocarcinogenesis by utilizing different animal models. The expression of DUSP1 was detected in two rat models: Fisher344 (F344) and Brown Norway(BN). Lower expression was detected in F344 rats which were susceptible to form chemical‐induced HCC, while BN rats expressed higher DUSP1 which were resistant to form HCC 30, 31. Susceptibility to hepatocarcinogenesis was identified to be associated with a more pronounced activation of the ERK cascade in F344 and BN rats. DUSP1, as an ERK inhibitor, played a role in inhibiting the hepatocarcinogenesis. Besides, other studies revealed that *Dusp1* co‐localizes with resistance locus *Hcr1* on chromosome 10, a region frequently affected by *LOH* during rat hepatocarcinogenesis 32. Genomic scanning analysis for rat hepatocarcinogenesis identified *Hcr1* as resistance loci 33. *Dusp1* may inhibit carcinogenesis in HCC by co‐operating with *Hcr1*. However, further study is necessary.

DUSP1 expression was also decreased in human head and neck squamous cell carcinoma (SCC) tissues compared with adjacent nontumorous controls. Recent studies revealed that *Dusp1*‐deficient mice were more susceptible to form SCC as a result of a liability to develop an inflammatory microenvironment in resopnse to carcinogen. DUSP1 was testified to inhibit SCC formation and mechanism study revealed DUSP1 decreased IL‐1*β* in tumor proinflammatory microenvironment to inhibit head and neck SCC formation 34, 35. Studying the relationship between DUSP1 and cancer‐associated inflammation provided a new perspective to explore its role in carcinogenesis (Table.1).

## Role of DUSP1 in Tumor Progression

Though DUSP1 plays a controversial role in carcinogenesis in different tumors, its role in tumor progression is relatively uniform (Table.1). In most cancers, such as prostate, ovarian, colon, and gastric cancer, progressive loss of DUSP1 is detected with histological grade increasing. DUSP1 mainly promotes carcinogenesis by inhibiting phosphorylation of JNK in these cancers; however, it mainly inhibits the tumor progression by inhibiting phosphorylation of ERK. Downregulating DUSP1 allows for proliferation and increased tumor mass in the more advanced stages of tumorigenesis by enhancing ERK/MAPK signaling pathway.

DUSP1 expression was also found to be significantly lower in HCC patients with poor prognosis than the patients with better prognosis 36, 37. However, Calvisi 36 found DUSP1 was not the main causative event responsible for phosphorylated ERK (p‐ERK) up‐regulation in human HCC. Besides, degradation of DUSP1 was detected in HCC via the ubiquitination proteasomal pathway caused by ERK2 activation 36, 38. The degradation of DUSP1 may then further strengthen the promotive effect on HCC growth by prolonging the half‐life of active ERK. Another study 39 indicated that p53 protein, as a tumor suppressor, suppresses cell growth by inducing cell cycle arrest or apoptosis via DUSP1. Recent study in HCC 37 revealed that wild‐type p53 could directly bind to DUSP1 gene and transcriptionally upregulated DUSP1. DUSP1 was involved in p53 activation via the p38 MAPK/HSP27 pathway. Downregulating the DUSP1 may interrupt the positive regulatory loop between DUSP1 and p53, and then promote HCC development and progression.

The role of DUSP1 in the progression of non‐small‐cell lung cancer (NSCLC) was inconclusive. Vicent's study 28 revealed that DUSP1 expression was a better prognosis factor in NSCLC. Higher DUSP1 expression in tumor nuclei staining was associated with a better survival. However, in another study, DUSP1 was positively related to tumor angiogenesis in NSCLC patients and there is a statistically significant correlation between VEGFC and DUSP1 expression in these patients. Furthermore, in vitro assay indicated that DUSP1 promotes angiogenesis, invasion, and metastasis in NSCLC cells 40. The reasons for these discrepancies on DUSP1 expression in NSCLS are still unknown. However, Vicent's study paid more attention to the DUSP1 expression in nuclei, and the subcellular localization of DUSP1 may need more considerations in future studies.

## Role of DUSP1 in Tumor Therapy

### DUSP1 and chemotherapy

**Figure 1:**
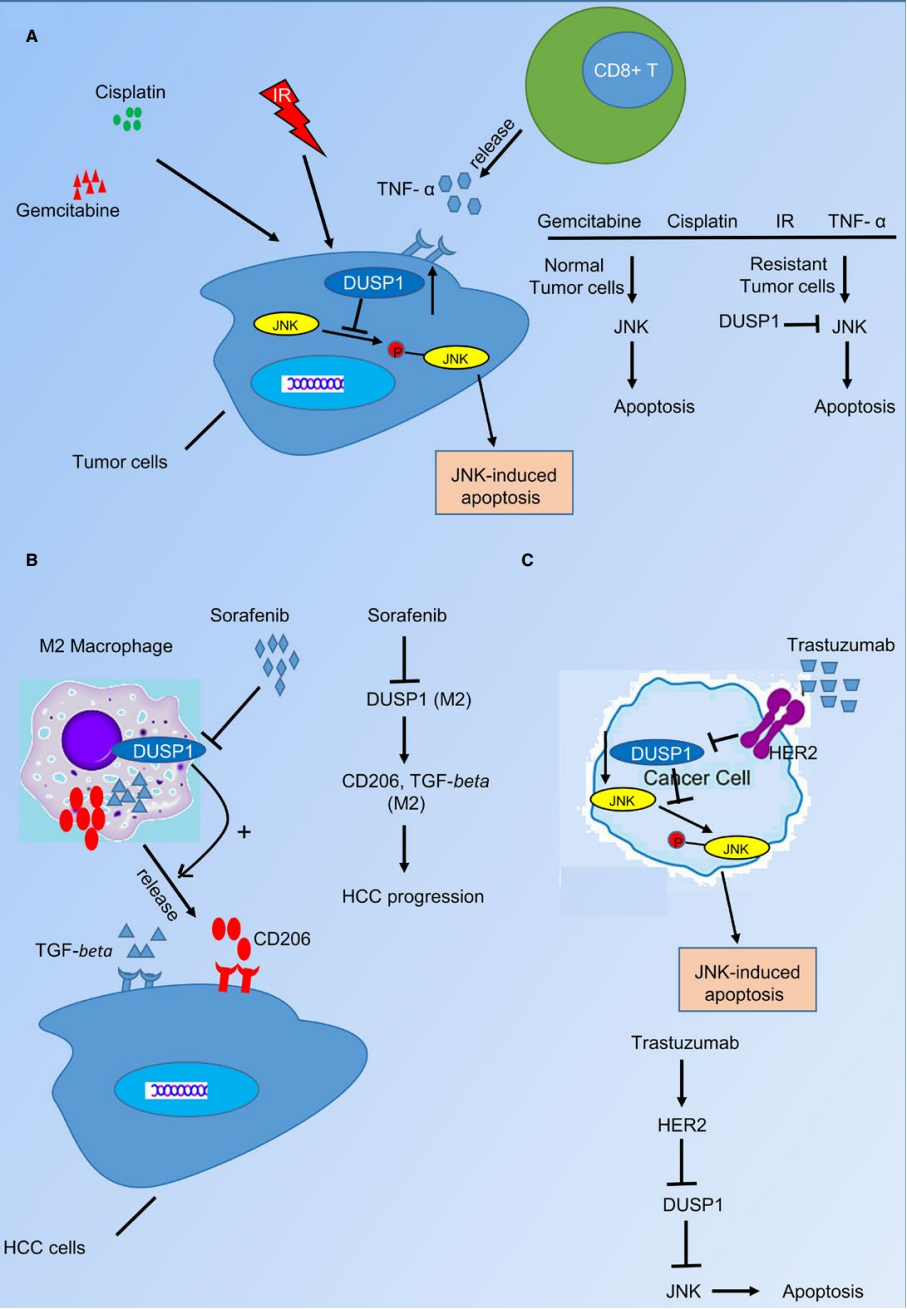
(A) Chemo‐drugs and radiation increased DUSP1 in tumor cells and decreased JNK‐induced apoptosis. (B) CD206 and TGF‐beta promoted HCC progression. Sorafenib inhibited HCC progression via decreasing DUSP1 in M2 macrophages and therefore decreased CD206 and TGF‐beta release. (C) Trastuzumad inhibited HER2 positive breast tumor cells via binding to HER2 to inhibit DUSP1 and therefore induced apoptosis in tumor cells. Trastuzumad‐resistant cells obtained a higher DUSP1 expression and abolished the drug effect.

An increase in DUSP1 levels was first found in the treatment of ovarian carcinoma cells with cisplatin. This suggests that DUSP1 may play a role in the cellular response to chemotherapy 41. Knocking down DUSP1 resulted in a more efficient activation of JNK and p38 under cisplatin treatment and correlated with an increase in sensitivity to cisplatin 42, 43, 44. DUSP1 was proved to be essential for cisplatin resistance as cells from *Dusp 1* knockout mice could not form resistance compared to normal cells 45. Besides, overexpressing DUSP1 also reduced sensitivity to cisplatin in NSCLC cells through the PI3K/Akt/NF‐kappaB pathway 46. Targeting DUSP1 also promoted dexamethasone sensitivity in lung cancer and gemcitabine sensitivity in pancreatic cancer 20, 47. DUSP1 promotes the chemoresistance of many chemo‐agents in various cancers (Fig. 1A).

### DUSP1 and radiation

DUSP1 was first found to play a protective role in U937 human leukemic cells against UV‐induced apoptosis by inhibiting UV‐induced SAPK activity 48. More recent work, using cells from the DUSP1 knockout mice, has demonstrated the protective role of DUSP1 in UV protection. Further study revealed that this entails crosstalk between the p38 pathway, which induces DUSP1 expression, and the JNK pathway, the inactivation of which, by DUSP1, prevents apoptosis 49. DUSP1 also plays a protective role in cancer cells after radiation. Nevertheless, DUSP1 affects JNK, p38 or extracellular signal‐regulated kinase subfamilies of MAPKs, inactivation of JNK, but not p38 or ERK‐abolished radiation‐induced proapoptotic status 50. DUSP1 was also reported to promote DNA repair process after radiation treatment by dephosphorylating H3S10^51^. In summary, elevated DUSP1 expression is negatively correlated with efficacy of radiotherapy. (Fig. 1A).

### DUSP1 and immunotherapy

By co‐culturing CD8^+^ T cells and M38 colorectal adenocarcinoma cell line, TNF‐ *α* released by CD8^+^ T cells was found to mainly mediate the cytotoxic effect (Fig. 1B). However, in M38 cells, DUSP1 blocks phosphorylation of JNK which is the key mediator of TNF‐ *α* receptor‐mediated cell death, and this in turn suppresses tumor cell death triggered by the immune system 52. This inhibition effect could be attenuated by knocking down DUSP1 in M38 cells which implies a protective role of DUSP1 in CD8^+^ T cells mediated cytotoxicity to cancer cells. DUSP1 plays an important role in this novel immune escape mechanism of tumors. The function of DUSP1 in other cancers' immune environment need to be further explored.

### DUSP1 and cancer molecular target drugs

Sorafenib is an effective and widely used molecular target drug in HCC 53, 54, 55, 56, 57. Besides the classical pathway directly targeting HCC, sorafenib was found to mediate the antitumor effect via macrophages. TGF‐beta and CD206 release from M2 cells was positively associated with hepatoma growth, metastases, and EMT 21, 58. Recent study revealed that sorafenib increased DUSP1 expression in M2 cells which leads to a lower TGF‐beta and CD206 release to better inhibit HCC progression. Interrupting DUSP1 in M2 cells could attenuate sorafenib's antitumor treatment. Studying the association between anticancer effects of sorafenib and elevated DUSP1 expression in M2 cells provides a new perspective to improve sorafenib's efficiency (Fig. 1C).

HER2, also known as ERBB2, was identified as an oncogene in breast cancer 59, 60, 61. HER2 is associated with poorer prognosis and has been applied as a common target for molecular therapy 62, 63, 64. The monoclonal antibody trastuzumab and the tyrosine kinase inhibitor lapatinib are currently clinical therapies targeting HER2 65, 66, however, adaptive resistance to anti‐HER2 therapy also occurrs in HER2‐positive breast cancer. Candas's study revealed a correlation between DUSP1 and HER2 expression in breast cancer. DUSP1 was detected as a key downstream target of HER2 and translocated into mitochondria where it prevents apoptotic induction by limiting accumulation of phosphorylated active forms of the stress kinase JNK 22. Trastuzumab decreased DUSP1 which leads to the accumulation of phosphorylated active forms of JNK to enhance apoptotic induction. However, DUSP1 was somehow found to be increased in anti‐HER2 therapy‐resistant cells and combined approaches interrupting both HER2 signaling and DUSP1 activity have proven even more successful [57].

## Summary and Future Prospects

DUSP1, as a phosphatase, could dephosphorylate two types of residues, threonine and tyrosine and therefore, inactivate the MAP kinases, ERKs, p38 MAPKs, and JNKs. It is not only involved in cellular proliferation, differentiation, and apoptosis but also the tumor carcinogenesis progression. DUSP1 plays different roles in different tumor stages and different cancers.

DUSP1 plays an important role in the carcinogenesis by targeting JNK‐induced apoptosis. It promotes carcinogenesis in various cancers including prostate, colon, bladder, gastric, breast, and lung cancer. However, it inhibits carcinogenesis in HCC by co‐operating with *Hcr1 and inhibits carcinogenesis in head and neck SCC by* decreasing IL‐1*β* in the inflammatory microenvironment. By targeting ERKs pathway and P53 pathway, DUSP1 also plays a positive role in the progression of most tumors, such as prostate, ovarian, colon, and gastric cancer. However, in lung cancer and head and neck SCC, the role of DUSP1 in tumor progression is still inconclusive. Besides, the role of DUSP1 in tumor therapy has been reviewed. The common approaches of tumor therapy includes: chemotherapy, radiation, immunotherapy, and biotherapy Figure 1. DUSP1 promotes resistance to chemotherapy and radiation in various cancers via decreasing JNK‐induced apoptosis 18, 20, 67, 68, 69. It also attenuates the cytotoxity of TNF‐*α* which was released by CD8^+^ T cells and decreases JNK‐induced apoptosis in cancer cells. Targeting DUSP1 enhances the efficiency of chemotherapy, radiation, and immune therapy. Developing molecular target drugs including sorafenib and trastuzumab is a new and promising perspective for antitumor treatment. However, drug resistance to these drugs also occurs. Combinatorial therapies interrupting both HER2 signaling and MKP1 activity have proven a better efficiency in treating HER2‐positive breast cancers. Targeting DUSP1 could overcome the impaired efficacy caused by drug resistance and significantly improve current antitumor drugs' activity. Further understanding of the different functions and mechanisms of DUSP1 in different tumors will facilitate its future applications as a novel therapeutic target.

## Conflict of Interest

The authors declare that there are no conflicts of interest.

## Supporting information


**Figure S1.** Protein structure of DUSP1.
