# Supplementary figures and images for: Role of DUSP1/MKP1 in tumorigenesis, tumor progression and therapy
Source: Cancer Med. 2016 May 26;5(8):2061–8. doi: 10.1002/cam4.772 (PMC4884638; doi:10.1002/cam4.772)

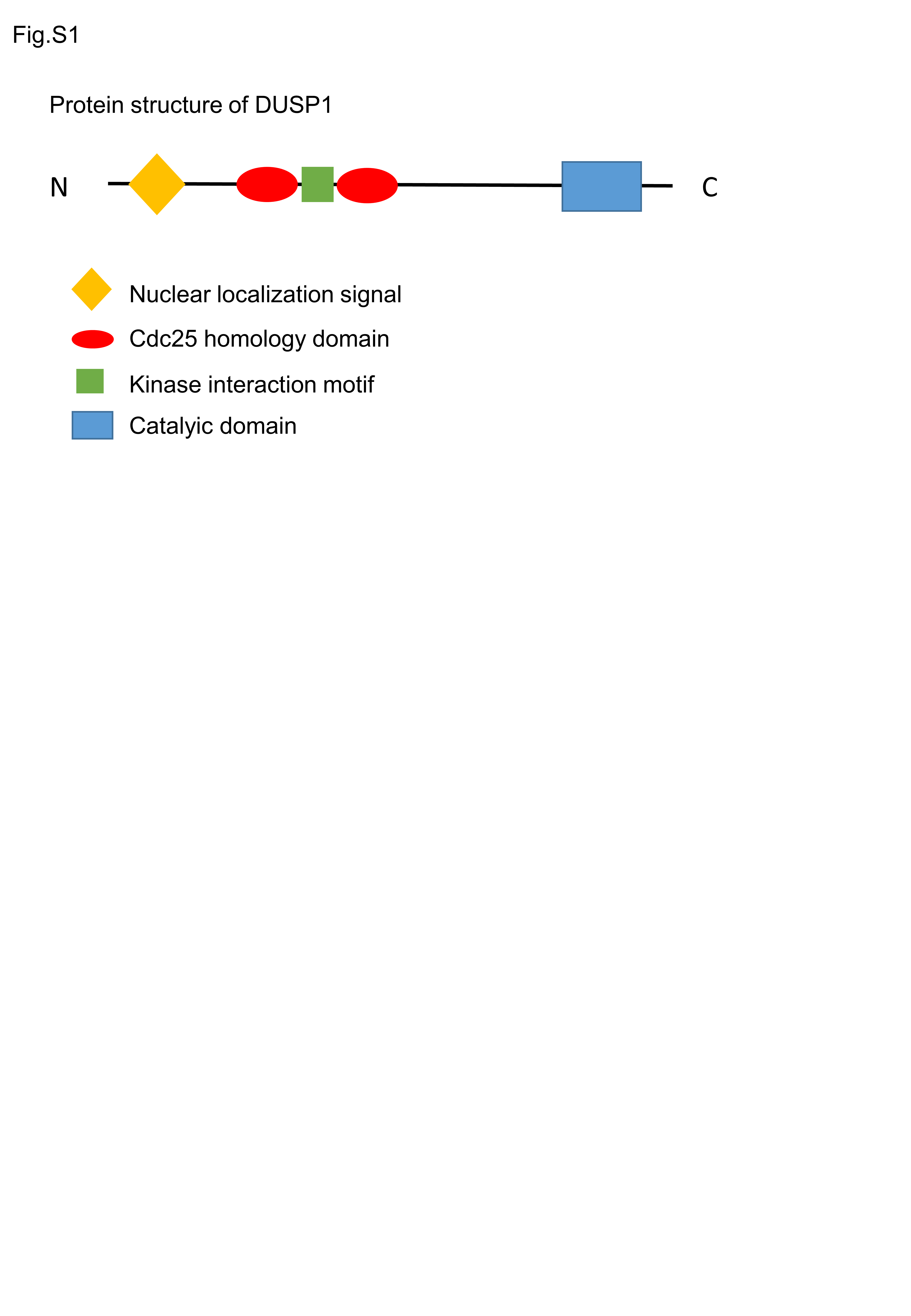

Supplement: Supplementary file 1 — Figure S1. Protein structure of DUSP1. [file CAM4-5-2061-s001.tif]
